# Enhanced trajectory tracking for autonomous navigation of wheeled mobile robots using an adaptive fuzzy PID controller

**DOI:** 10.1038/s41598-026-45772-y

**Published:** 2026-04-18

**Authors:** Helmy M. El Zoghby, Soliman M. Sharaf, Ahmed F. Bendary, Ahmed Hessien

**Affiliations:** Electrical Power and Machines Engineering, Faculty of Engineering, Helwan University, Helwan, Egypt

**Keywords:** Mobile robots, Trajectory tracking, PID controller, Fuzzy logic controller, Engineering, Mathematics and computing

## Abstract

With the advancement of autonomous technologies, the need for robust control strategies in unstructured environments is becoming increasingly important. The ability to track a trajectory and accurately control the motion of a robot is a key aspect of mobile robotics and is essential if wheeled mobile robots (WMRs) are to successfully perform tasks and function in the real world. Due to the complex and unstructured working environments, the control systems of WMRs must deal with difficulties such as extreme non-linear behaviors of the dynamic systems, unmodeled parameters of the systems, and external disturbances. This paper presents an adaptive fuzzy gain scheduling PID controller that addresses the challenges posed by structured uncertainties like kinematic wheel slips, random actuator noise, and external disturbances. The controller is based on a robust cascaded control structure that can be used for tracking the desired trajectory. The robustness of the controller is ensured by uniformly ultimately bounded analysis. The control system incorporates adaptive fuzzy logic and PID control to achieve a more advanced level of trajectory-tracking control. The control systems enhance fuzzy logic and PID control with an adaptive capability aimed at improving the systems’ robustness against external disturbances. Changes in the robot dynamics or the environment may happen, but the controller can adjust its parameters in real time and achieve the desired result due to the controller’s adaptation mechanism. The efficacy of the controller is validated using extensive simulations for tracking complex lemniscate ($\infty$) curves. The results are compared with conventional PID and adaptive dynamic control methods to demonstrate that the proposed controller reduces the RMS tracking error. The results clearly demonstrate that the controller can reject disturbances while achieving precise navigation even for 100% variations in the parameters. The results presented in this paper provide a computationally efficient framework to bridge the gap between kinematics and dynamics for real-time robotic applications.

## Introduction

Unmanned ground vehicles (UGVs) have become more common in industrial and scientific settings recently^1,2^. Among these, autonomous mobile robots (AMRs) have attracted a lot of interest in a variety of fields, such as industry, entertainment, services, and hazardous situations. AMRs can now carry out a variety of complex activities in both daily life and industrial contexts thanks to advancements in sensor, microprocessor, and control technology. An autonomous mobile robot must successfully perceive, decide, and act in order to navigate a certain area safely and accurately. Their widespread use is largely due to their energy economy, fast maneuverability, steady operation, and straightforward control methods. The creation of robust and intelligent autonomous controllers is essential to accomplish given tasks with low mistakes across a variety of AMR domains, including service, agriculture, and industries, especially in human-inaccessible places like space exploration or high-risk nuclear installations^3^.

Wheeled mobile robots (WMRs) and legged robots are two basic categories of UGVs based on their manner of propulsion. Compared to legged robots, WMRs are typically less expensive and easier to build and operate. Path planning, trajectory building, trajectory control, navigation, and stability are the main research topics in WMRs. In particular, creating a control law that allows a WMR to perfectly follow a predetermined reference path with particular time parameters is the trajectory tracking problem. Such a tracking controller’s primary goal is to reduce tracking errors by ensuring the WMR precisely follows the specified trajectory while preserving a reference velocity. In spite of measurement noise, uncertainty, and outside disruptions, an effective controller must produce the desired performance.

In the past few years, researchers have created a variety of trajectory tracking control techniques. To solve trajectory tracking issues, a variety of control strategies have been used, frequently including adaptive feedback systems that depend on a robot’s dynamic model. For example, Lyapunov stability theory was used in^4^ to create a robust adaptive controller for AMR trajectory tracking. In^5^, tracking the trajectory for a mobile autonomous robot was addressed using a conventional PID controller with a unique parameter tuning technique, guaranteeing closed-loop system stability. Two Takagi–Sugeno (TS) model-driven fuzzy blocks were used in^6^ to modify the scale factors of a fuzzy controller designed for robot tracking of a trajectory that was based on the stability of Lyapunov theory. Reference^7^ posed a WMR trajectory tracking control problem with wheel slippage and skid under a logical control pattern for both longitudinal and lateral slip components. Nonlinear model predictive control (MPC) for visual trajectory tracking control was suggested in^8^ with the goal of computing control commands on the image plane. In^9^ the tracking error learning control (TELC) scheme was proposed for the problem of mobile robots’ trajectory following and utilising the tracking error dynamics for updating control action feedforward corrections. Reference^10^ presents a design of an adaptive dynamic controller for a differential mobile robot motion control by means of differential dynamic model. For the case of differential mobile robots, backstepping path tracking with fuzzy sliding mode control was constructed in^11^, where a fuzzy controller was used to mitigate the chattering phenomenon and the results were compared with a classical backstepping sliding mode controller. Additionally, in^12^, a novel nonlinear observer and an adaptive model reference technique were employed for an adaptive path-tracking control scheme for WMRs without velocity sensors. Artificial immunity was utilising in^13^ to enhance a fuzzy controller for path tracking of a mobile wheeled robot, comparing it against a standard fuzzy controller. Based on the kinematics model and artificial intelligence theory, the adaptive path tracking controller in^14^ addressed the non-holonomic constraint problem in robot trajectory tracking. To ensure stability and system performance against disturbances and parameter changes, Wang et al.^15^ implemented a two-degree-of-freedom dynamic model with an adaptive PID controller to formulate the path-tracking problem for an AMR in state-space format.

Most dynamic controllers described in the literature typically produce control signals in the form of torques or motor voltages for the robots (as seen in the aforementioned publications). However, commercial robots, such as the RobuLAB-10, Pioneer, and Khepera series, commonly accept velocity commands. In order to overcome this, Zhao et al.^16^ created a hierarchical switching controller with online learning and neural network-based methods to counteract unmodelled events. However, for real-time deployment, their approach needed a high-performance multiprocessor system. On the other hand, Antonini et al.^17^ suggested a dynamic model that benefits from parameters directly related to the physical characteristics of the robot by using angular and linear velocities as inputs in conjunction with a multi-robot controller design. Expanding on this idea, dynamic models with linear and angular velocities as inputs have been used in a number of research studies. For trajectory-tracking missions, an adaptive sliding-mode dynamic controller was presented in^18^. This controller combines a kinematic controller to guarantee that the WMR’s real velocity corresponds with the intended velocity commands. In^19^, a landmark-based navigation system for robotic wheelchairs was constructed in conjunction with an adaptive controller that took into account its dynamic model. De La Cruz et al.^20^ introduced an online learning integration of inverse nonlinear control, an adaptive neural network, with sliding mode control for mobile robot trajectory tracking. Here, an adaptive NN serves as a compensator to improve overall system performance by compensating for the controller changes. Rossomando et al.^21^ discussed the nonlinear model predictive controller of an agricultural robot operating in row crops to maintain an accurate trajectory for the high-precision, drop-on-demand herbicide spraying.

The dynamics of WMR and its controllers are and always will be complicated and nonlinear as a problem. With regard to trajectory tracking, a divergence of controllers, both linear and nonlinear, has been proposed. However, linear controllers, i.e., PID and LQR, are more dominant due to their simplistic structure, less demanding computational power, and overall satisfactory performance. Still, their systems only provide adequate results when operating at the designated operating point, as noted in^22^, and classical PID controllers do not guarantee global stability^23^.

Despite the fact that nonlinear saturation functions have been suggested as a means of achieving global stability, their effectiveness frequently declines dramatically in the face of disruptions, poor sensor data, and model errors. Variations in plant characteristics, model uncertainties, and outside disruptions are additional significant obstacles in the design of mobile robot tracking controllers. An adaptive nonlinear controller can provide better and desired tracking performance because it does not require linearised approximation and allows for online control parameter tweaking. Because of their strong learning skills and ability to simulate any nonlinear system, neural networks are becoming more and more common in adaptive controller parameter tuning^24^.

This work focuses on creating an adaptive fuzzy logic controller (AFLC) to address the trajectory-following control problem of a wheeled mobile robot, driven by the restrictions indicated above and the potential of adaptive nonlinear control. This controller is specifically made to function well even in the face of external disturbances, parameter uncertainties, and measurement noises. The developed control technique applies to WMRs used in factories, warehouses, missions to space, surveillance, and other industries, providing satisfactory performance under a variety of challenging settings.

This study makes the following primary contributions:

- Robust disturbance rejection capability: One significant contribution is the incorporation of external disturbances into simulation scenarios. The suggested ST-FPID is exceptionally robust in preserving trajectory accuracy even when subjected to external loads and environmental noise, effectively “rejecting” these perturbations better than traditional methods.
- Adaptive control under uncertainty: The creation of an adaptive scheme that accounts for model uncertainty and parameter variations. The controller dynamically recalibrates its gains in real time, ensuring that the robot adheres to the established trajectory with minimal tracking error independent of internal or external variances.
- Optimized multi-output fuzzy architecture: Creating a streamlined fuzzy logic architecture with two inputs and three outputs. This approach uses relational logic to reduce linguistic concepts, reducing computational cost while allowing for simultaneous, optimal tuning of *k*_*p*_, *k*_*i*_, and *k*_*d*_.
- Superior performance metrics: The ST-FPID outperforms traditional PID, according to a rigorous comparison study. It achieves faster convergence and much lower distance error, notably in the high curvature regions of the Lemniscate trajectory.
- Comprehensive testing scenarios: Evaluate the control system in many simulations, ranging from perfect conditions to more tough situations with added noise, parameter fluctuation, and disruptions. The quantitative results (RMSE, IAE and ITAE) consistently demonstrate the better efficacy and dependability of the proposed framework.

## Modelling of differential-drive mobile robot

### Kinematic model of the mobile robot

**Fig. 1:**
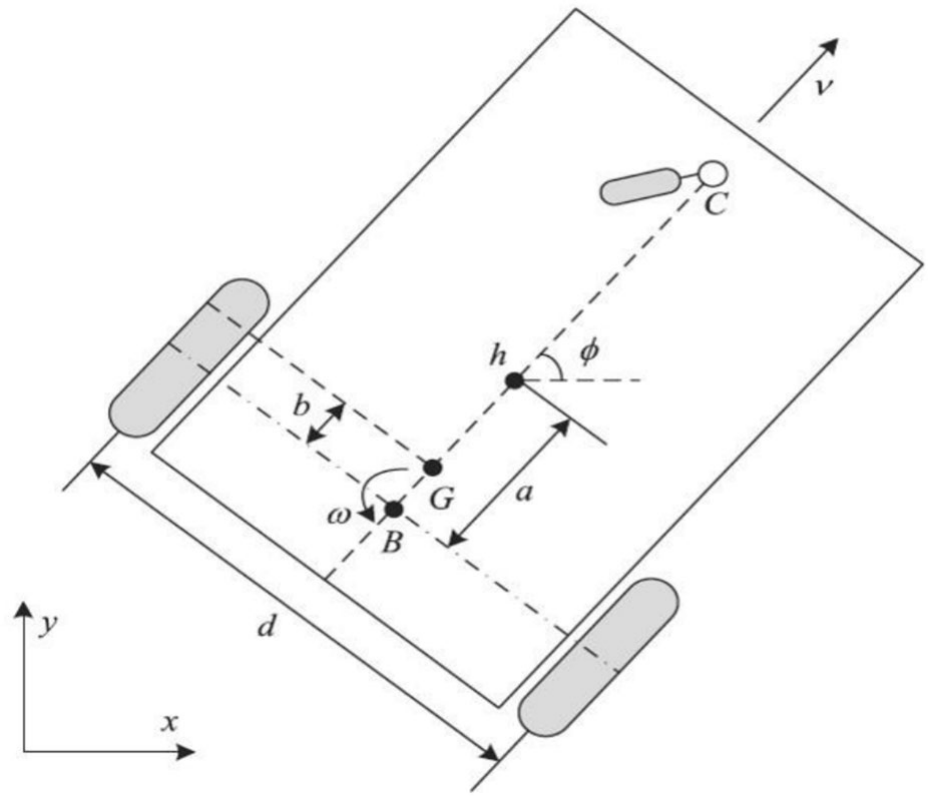
Model of a typical differential-drive mobile robot.

The design of a trajectory tracking controller for a differential-drive mobile robot (DDMR) is examined in this work. Figure 1 shows a sketch of the DDMR model, and we assume that the mobile robot’s workspace is an ideal plane (Table 1).

**Table 1:** Physical and geometric parameters of the mobile robot.

Symbol	Description
*d*	Distance between the two driving wheels
*a*	Longitudinal distance from the mid-axis *B* to the point of interest *h*
*b*	Offset distance of the center of mass *G* from the driving axis
${\emptyset}$	Orientation (heading angle) of the robot with respect to the X-axis
*v*	Linear velocity of the robot
*ω*	Angular velocity of the robot
*G*	Center of mass of robot
*B*	Reference point at the center of the driving wheels’ axis
*h*	The central point of axis connecting of two wheels
*m*	Mass
*I*_*z*_	The moment of inertia at *G*
*R*_*a*_	The electric resistance of its motors
*K*_*b*_	The electromotive constant of its motor
*K*_*a*_	The constant of torque of its motor
*B*_*e*_	The coefficient of friction
*I*_*e*_	The moment of inertia of each group rotor-reduction gear-wheel
*r*	The radius of the wheels

The mobile robot moving in two dimensions this is my consideration, shown in Fig. 1.

1 $$P = \left[ {\begin{array}{*{20}c} x \\ y \\ \emptyset \\ \end{array} } \right]$$

The position of mobile robot XOY initial reference frame.

2 $$P{\mathrm{i}}\left[ {\begin{array}{*{20}c} {xi} \\ {yi} \\ {\emptyset {\mathrm{i}}} \\ \end{array} } \right]$$

The interaction between the robot frame and global frame can be illustrated as:

3 $$P = R\left( \theta \right)P{\mathrm{i}}$$

where

$$R\left( \theta \right) = \left[ {\begin{array}{*{20}c} {cos\, \emptyset } & {sin\, \emptyset } & 0 \\ { - sin\, \emptyset } & {cos\, \emptyset } & 0 \\ 0 & 0 & 1 \\ \end{array} } \right]$$

The study of a robot’s motion without taking into account the forces causing it is known as kinematics. A mobile robot’s forward kinematics can be found as

4 $$\left[ {\begin{array}{*{20}c} {\dot{x}} \\ {\dot{y}} \\ {\dot{\theta }} \\ \end{array} } \right] = \left[ {\begin{array}{*{20}c} {v \, cos\, \emptyset } \\ {v \, sin\, \emptyset } \\ \omega \\ \end{array} } \right]$$

Then the mobile robot kinematic model is derived as:

5 $$\left[ {\begin{array}{*{20}c} {\dot{\mathrm{x}}} \\ {\dot{\mathrm{y}}} \\ {\dot{\theta }} \\ \end{array} } \right] = \left[ {\begin{array}{*{20}c} {\cos \emptyset } & { - a\sin \emptyset } \\ {\sin \emptyset } & {a\cos \emptyset } \\ 0 & 1 \\ \end{array} } \right]\left[ {\begin{array}{*{20}c} v \\ \omega \\ \end{array} } \right]$$

### Dynamic model

The study of a mobile robot’s mobility while taking into account the forces causing it is known as dynamics. Since the WMR dynamic model is more complex, most motion control algorithms are designed using the kinematic model. However, if the applications call for large payloads and rapid movements, a dynamic model is unavoidable. Odometry is used by the majority of autonomous mobile robots to localize their positions during navigation. Odometric error accumulation from slip at the wheel-ground contact sites might impair the functionality of sensor-based navigation algorithms.

To increase navigation performance, wheel-slip information should be taken into account while designing WMR models. Most practical applications do not meet the no-slip condition assumption. Controlling the slip magnitude can increase the mobile robots’ maneuverability. As a result, in this work, the dynamics of a slide are integrated into a WMR model. This study discusses the mathematical model, which includes the kinematic and dynamic models that involve the slip dynamics, as presented in^12,16^ and defined as

6 $$\left[ {\begin{array}{*{20}c} {\dot{\mathrm{x}}} \\ {\dot{\mathrm{y}}} \\ {\begin{array}{*{20}c} {\dot{\emptyset }} \\ {\begin{array}{*{20}c} {\dot{v}} \\ {\dot{\omega }} \\ \end{array} } \\ \end{array} } \\ \end{array} } \right] = \left[ {\begin{array}{*{20}c} {u\, cos\, \emptyset - aw\, sin\, \emptyset } \\ {u\, sin\, \emptyset + aw\, sin\, \emptyset } \\ {\begin{array}{*{20}c} \omega \\ {\frac{{\delta_{3} }}{{\delta_{1} }}\omega^{2} - \frac{{\delta_{4} }}{{\delta_{1} }}u} \\ {\frac{{\delta_{5} }}{{\delta_{2} }}u\omega - \frac{{\delta_{6} }}{{\delta_{2} }}\omega } \\ \end{array} } \\ \end{array} } \right] + \left[ {\begin{array}{*{20}c} {\begin{array}{*{20}c} 0 \\ {\begin{array}{*{20}c} 0 \\ {\begin{array}{*{20}c} 0 \\ {\frac{1}{{{ \upgamma }_{1} }}} \\ 0 \\ \end{array} } \\ \end{array} } \\ \end{array} } & {\begin{array}{*{20}c} 0 \\ {\begin{array}{*{20}c} 0 \\ {\begin{array}{*{20}c} 0 \\ 0 \\ {\frac{1}{{{ \upgamma }_{2} }}} \\ \end{array} } \\ \end{array} } \\ \end{array} } \\ \end{array} } \right]\left[ {\begin{array}{*{20}c} {u_{{\mathrm{r}}} } \\ {\omega_{{\mathrm{r}}} } \\ \end{array} } \right] + \left[ {\begin{array}{*{20}c} {\zeta_{{\mathrm{x}}} } \\ {\zeta_{y} } \\ {\begin{array}{*{20}c} 0 \\ {\zeta_{v} } \\ {\zeta_{\omega } } \\ \end{array} } \\ \end{array} } \right]$$

where $u_{{\mathrm{r}}}$ and $\omega_{{\mathrm{r}}}$ are the reference linear and angular velocities, the mathematical equations that define the parameters $\delta_{i}$ are^10^:

7 $$\begin{aligned} \delta _{1} & = \left[ {\frac{{{\mathrm{R}}_{{\mathrm{a}}} }}{{{\mathrm{k}}_{{\mathrm{b}}} }}\left( {{\mathrm{mr}}^{2} + 2{\mathrm{I}}_{{\mathrm{e}}} } \right) + 2{\mathrm{rk}}_{{{\mathrm{DT}}}} } \right]\frac{1}{{\left( {2{\mathrm{rK}}_{{{\mathrm{PT}}}} } \right)}}\, \left[ {\mathrm{s}} \right], \\ \delta _{2} & = \left[ {\frac{{{\mathrm{R}}_{{\mathrm{a}}} }}{{{\mathrm{k}}_{{\mathrm{a}}} }}\left( {{\mathrm{I}}_{{\mathrm{r}}} {\mathrm{d}}^{2} + 2{\mathrm{r}}^{2} \left( {{\mathrm{I}}_{{\mathrm{z}}} + {\mathrm{mb}}^{2} } \right)} \right) + 2{\mathrm{rdk}}_{{{\mathrm{DR}}}} } \right] \times \frac{1}{{\left( {2{\mathrm{rdk}}_{{{\mathrm{PR}}}} } \right)}}\,\left[ {\mathrm{s}} \right], \\ \delta _{3} & = \frac{{{\mathrm{R}}_{{\mathrm{a}}} }}{{{\mathrm{k}}_{{\mathrm{a}}} }}\frac{{{\mathrm{mbr}}}}{{2{\mathrm{K}}_{{{\mathrm{PT}}}} }}\left[ {{\mathrm{sm/rad}}^{2} } \right], \\ \delta _{4} & = \frac{{{\mathrm{R}}_{{\mathrm{a}}} }}{{{\mathrm{k}}_{{\mathrm{a}}} }}\left( {\frac{{{\mathrm{k}}_{{{\mathrm{ak}}_{{\mathrm{b}}} }} }}{{{\mathrm{R}}_{{\mathrm{a}}} }} + {\mathrm{B}}_{{\mathrm{e}}} } \right)\frac{1}{{{\mathrm{rK}}_{{{\mathrm{PT}}}} }} + 1, \\ \delta _{5} & = \frac{{{\mathrm{R}}_{{\mathrm{a}}} }}{{{\mathrm{k}}_{{\mathrm{a}}} }}\, \frac{{{\mathrm{mbr}}}}{{{\mathrm{dk}}_{{{\mathrm{PR}}}} }}\;\left[ {{\mathrm{s/m}}} \right],\;{\mathrm{and}} \\ \delta _{6} &= \frac{{{\mathrm{R}}_{{\mathrm{a}}} }}{{{\mathrm{k}}_{{\mathrm{a}}} }}\left( {\frac{{{\mathrm{k}}_{{{\mathrm{ak}}_{{\mathrm{b}}} }} }}{{{\mathrm{R}}_{{\mathrm{a}}} }} + {\mathrm{B}}_{{\mathrm{e}}} } \right)\frac{{\mathrm{d}}}{{\left( {2{\mathrm{rk}}_{{{\mathrm{PR}}}} } \right)}} + 1. \\ \end{aligned}$$

It should be clarify that $\delta_{i} > 0$ for i = 1, 2, 4, 6. The parameters $\delta_{3}$ and $\delta_{5}$ can be negative and will be null if, and only if the center of mass G is exactly in the center of the virtual axle, i.e. b = 0.

The above model is split into kinematic and dynamic parts.

The kinematic model with disturbance $\zeta_{kin}$:

8 $$\left[ {\begin{array}{*{20}c} {\dot{\mathrm{x}}} \\ {\dot{\mathrm{y}}} \\ {\dot{\theta }} \\ \end{array} } \right] = \left[ {\begin{array}{*{20}c} {cos\emptyset } & { - dsin\emptyset } \\ {\begin{array}{*{20}c} {sin\emptyset } \\ 0 \\ \end{array} } & {\begin{array}{*{20}c} {dcos\emptyset } \\ 1 \\ \end{array} } \\ \end{array} } \right]\left[ {\begin{array}{*{20}c} u \\ \omega \\ \end{array} } \right] + \left[ {\begin{array}{*{20}c} {\zeta_{\mathrm{x}} } \\ {\zeta_{y} } \\ 0 \\ \end{array} } \right]$$

To account for the non-ideal constraints of the mobile robot, the kinematic model is augmented with a disturbance vector $$\zeta_{kin} = \left[ {\begin{array}{*{20}c} {\zeta_{x} } & {\zeta_{y } } \\ \end{array} 0} \right]^{T}$$. Physically, these terms represent kinematic uncertainties arising from wheel slipping and skidding, which cause deviations between the commanded velocities and the actual robot trajectory. In the simulation, this effect is modeled as a stochastic process to evaluate the controller’s ability to maintain precise path tracking despite the violation of pure rolling conditions.

Whereas the dynamic model with disturbance $\zeta_{{{\mathrm{dyn}}}}$:

9 $$\left[ {\begin{array}{*{20}c} {\dot{v}} \\ {\dot{\omega }} \\ \end{array} } \right] = \left[ {\begin{array}{*{20}c} { \frac{{\delta_{3} }}{{\delta_{1} }}\omega^{2} - \frac{{\delta_{4} }}{{\delta_{1} }}u} \\ { - \frac{{\delta_{5} }}{{\delta_{2} }}u\omega - \frac{{\delta_{6} }}{{\delta_{2} }}\omega } \\ \end{array} } \right] + \left[ {\begin{array}{*{20}c} {\frac{1}{{\delta_{1} }}} & 0 \\ 0 & {\frac{1}{{\delta_{2} }}} \\ \end{array} } \right]\left[ {\begin{array}{*{20}c} {{u}_{\mathrm{r}}} \\ {{\omega }_{\mathrm{r}}} \\ \end{array} } \right] + \left[ {\begin{array}{*{20}c} {\zeta_{v} } \\ {\zeta_{\omega } } \\ \end{array} } \right]$$

The dynamic model incorporates a lumped disturbance term $$\zeta_{dyn} = \left[ {\begin{array}{*{20}c} {\zeta_{v} } & {\zeta_{w } } \\ \end{array} } \right]^{T}$$ added to the control input channels. This vector encapsulates unmodeled dynamics, such as actuator jitter, nonlinear friction, and battery voltage fluctuations. By implementing this disturbance as a band-limited white noise with a power of 0.1, we demonstrate the robustness and disturbance rejection capabilities of the proposed control law, ensuring its reliability under high-frequency torque perturbations typical in real-world hardware.

## Synthesis of the control law

To regulate the motion of the mobile robot, a kinematic-based control strategy with self-tuning fuzzy PID controllers is designed in this section. The kinematic layer of the control structure is responsible for correcting position errors, whereas the dynamic layer employs fuzzy logic-based PID controllers to monitor the velocity of the driving wheels. The fuzzy logic controller is employed in the dynamic layer to dynamically adjust the PID parameters ($k_{p} ,k_{i} ,k_{d}$) in order to ensure tracking reliability.

### Kinematic controller

The kinematic controller is designed based on the robot’s kinematic model, with the assumption that the disturbance term in (6) is zero. The robot’s kinematic model can be calculated using (6):

10 $$\left[ {\begin{array}{*{20}c} {\dot{\mathrm{x}}} \\ {\dot{\mathrm{y}}} \\ {\dot{\theta }} \\ \end{array} } \right] = \left[ {\begin{array}{*{20}c} {\cos \emptyset } & { - a\sin \emptyset } \\ {\sin \emptyset } & {a\cos \emptyset } \\ 0 & 1 \\ \end{array} } \right]\left[ {\begin{array}{*{20}c} v \\ \omega \\ \end{array} } \right]$$

The result is the coordinates of the location of interest, which means $$P = \left[ {\begin{array}{*{20}c} x & y \\ \end{array} } \right]^{T}$$.

11 $$P = \left[ {\begin{array}{*{20}c} {\dot{x}} \\ {\dot{y}} \\ \end{array} } \right] = \left[ {\begin{array}{*{20}c} {\cos \emptyset } & { - a\sin \emptyset } \\ {\sin \emptyset } & {a\cos \emptyset } \\ \end{array} } \right]\left[ {\begin{array}{*{20}c} v \\ \omega \\ \end{array} } \right] = q\left[ {\begin{array}{*{20}c} v \\ \omega \\ \end{array} } \right]$$

$${{q}} = \left[ {\begin{array}{*{20}c} {\cos \emptyset } & { - {{a}}\sin \emptyset } \\ {\sin \emptyset } & {{{a}}\cos \emptyset } \\ \end{array} } \right]$$

The inverse of ${{q}}$

12 $${{q}}^{ - 1} = \left[ {\begin{array}{*{20}c} {\cos \emptyset } & {\sin \emptyset } \\ { - \frac{1}{{{a}}}\sin \emptyset } & {\frac{1}{{{a}}}\cos \emptyset } \\ \end{array} } \right]$$

So, the inverse kinematics is given by

13 $$\left[ {\begin{array}{*{20}c} {{v}} \\ {{\omega}} \\ \end{array} } \right] = \left[ {\begin{array}{*{20}c} {\cos \emptyset } & {\sin \emptyset } \\ { - \frac{1}{{{a}}}\sin \emptyset } & {\frac{1}{{{a}}}\cos \emptyset } \\ \end{array} } \right]\left[ {\begin{array}{*{20}c} {\dot{{x}}} \\ {\dot{{y}}} \\ \end{array} } \right]$$

The kinematic control law for robots is as follows:

14 $$\left[ {\begin{array}{*{20}c} {{{v}}_{{{r}}}^{{{c}}} } \\ {{{{\upomega}}}_{{{r}}}^{{{c}}} } \\ \end{array} } \right] = \left[ {\begin{array}{*{20}c} {\cos \emptyset } & {\sin \emptyset } \\ { - \frac{1}{{{a}}}\sin \emptyset } & {\frac{1}{{{a}}}\cos \emptyset } \\ \end{array} } \right]\left[ {\begin{array}{*{20}c} {\dot{\mathrm{x}}_{{\mathrm{d}}} + {{l}}_{{\mathrm{x}}}\, {{tanh}}\left( {\frac{{\begin{array}{*{20}c} {{{k}}_{{{x}}} } \\ \end{array} }}{{\begin{array}{*{20}c} {{{l}}_{{{x}}} } \\ \end{array} }}\tilde{{x}}} \right)} \\ {\dot{\mathrm{y}}_{{\mathrm{d}}} + {{l}}_{{{y}}}\, {{tanh}}\left( {\frac{{{{k}}_{{{y}}} }}{{{{l}}_{{{y}}} }}\tilde{{y}}} \right)} \\ \end{array} } \right]$$

where the saturation constant are $${\mathrm{l}}_{{\mathrm{x}}}$$ and $${\mathrm{l}}_{{\mathrm{y}}}$$, the control output of kinematic controller are ($$v_{r}^{c} )$$ linear velocity and $$({\upomega }_{r}^{c} )$$ angular velocity, $${\mathrm{a}} > 0$$. The actual position error in the axis ($$x,y$$) are $${\tilde{\mathrm{x}}}$$ = $$x_{d} - x$$, $$\tilde{y} = y_{d} - y$$; $${\mathrm{k}}_{{\mathrm{x}}} > 0$$ and $${\mathrm{k}}_{{\mathrm{y}}} > 0$$ are the gains of controller and ($$x,y$$) and $$(x_{d} ,y_{d} )$$ are the current and the desired location of robot. This controller generates references for linear and angular velocities used in dynamic control.

### Dynamic controller adaptive fuzzy PID controller

#### Structure of the fuzzy PID controller

**Fig. 2:**
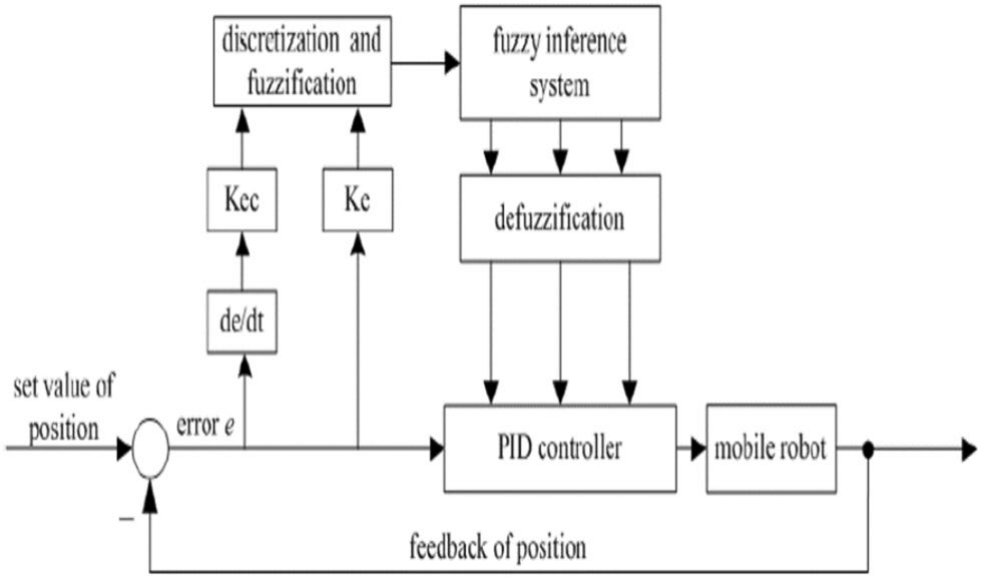
The structure of the proposed control system.

The proposed control strategy employs a cascaded hierarchical architecture. The kinematic layer processes the posture error to generate the reference velocity vector ($$V_{r} = \left[ {\begin{array}{*{20}c} {v_{r}^{c} } & {\omega_{r}^{c} } \\ \end{array} } \right]^{T}$$). These signals act as the input set-points for the dynamic layer, which is governed by a fuzzy gain-scheduling (FGS) PID controller. The mapping between these layers is defined as ($$U_{PID} = \left[ {\begin{array}{*{20}c} {u_{r} } & {\omega_{r} } \\ \end{array} } \right]$$), where $u_{r}$ and $\omega_{r}$ represent the linear and angular control efforts required to achieve the desired motion. The structure of the control system shown in Fig. 2.

The primary challenge in fuzzy PID system design is determining the proper scheduling parameter^9^. Fuzzy logic is designed to schedule and modify PID controller parameters to eliminate errors at the desired level^8^. In the design of fuzzy-PID logic control based on the Mamdani reasoning technique, a fuzzy set applied to the system output uses a triangular rule basis (trim) to identify the desired output. A fuzzy system with two input errors (*e* and change of error (*Ce*)) and three outputs (${\mathrm{k}}_{{{\mathrm{p}}1}} ,{\mathrm{k}}_{{{\mathrm{i}}1}} ,$ and ${\mathrm{k}}_{{{\mathrm{d}}1}}$) is shown in Fig. 2.

The fuzzy-PID logic control system is a closed-loop system that deals with a mobile robot’s rotational velocity set point (rpm). This research uses error variables and their changes as two inputs to the fuzzy logic controller. When the current error value is compared to the previous error value, the error change is calculated^10^. The PID input is *e*, and a $U_{PID}$ will be produced by the PID control’s output control after it is tuned.

15 $$U_{PID} = k_{p2} e\left( t \right) + k_{i2} \mathop \int \limits_{0}^{t} e\left( t \right)dt + k_{d2} \frac{de\left( t \right)}{{dt}}$$

$$\begin{aligned} & k_{p2} \left( t \right) = k_{p} \times k_{p1} \left( {e,Ce} \right) \\ & k_{i2} \left( t \right) = k_{i} \times k_{i1} \left( {e,Ce} \right) \\ & k_{d2} \left( t \right) = k_{d} \times k_{d1} \left( {e,Ce} \right) \\ \end{aligned}$$

The tracking error is defined as:

$$e\left( t \right) = r\left( t \right) - y\left( t \right)$$

The change of error is defined as:

$$Ce\left( t \right) = de\left( t \right)/d\left( t \right)$$

The control system integrates a fuzzy logic component with a classical PID controller. Initially, the PID controller operates with a predefined set of parameters: ($k_{p} ,k_{i} ,k_{d}$). The fuzzy inference engine, which continuously fine-tunes these parameters using a rule-based approach, is the central component of our adaptive technique. The fuzzy reasoning process adapts the controller to the dynamic behavior of the system by producing a new, modified set of PID parameters, known as ($k_{p1} ,k_{i1} ,k_{d1}$), based on a set of “if–then” fuzzy rules.

### Stability analysis

The main control objective of this study is to guarantee that the autonomous system tracks the time-varying reference trajectory given by $$q_{d} \left( t \right) = \left[ {\begin{array}{*{20}c} {x_{r} } & {y_{r} } & {\emptyset_{r} } \\ \end{array} } \right]^{{T}}$$. To monitor the performance, the tracking error vector e(t) is defined in the local coordinate system as follows:

16 $${{e}}\left( t \right) = \left[ {\begin{array}{*{20}c} {{\mathrm{e}}_{{{x}}} } \\ {{{e}}_{{{y}}} } \\ {{{e}}_{\emptyset } } \\ \end{array} } \right] = \left[ { - \begin{array}{*{20}c} {\cos \emptyset } & {\sin \emptyset } & 0 \\ {\sin \emptyset } & {\cos \emptyset } & 0 \\ 0 & 0 & 1 \\ \end{array} } \right]\left[ {\begin{array}{*{20}c} {{{x}}_{{{r}}} - {{x}}} \\ {{{y}}_{{{r}}} - {{y}}} \\ {\emptyset_{{{r}}} - \emptyset } \\ \end{array} } \right]$$

Despite the presence of nonlinearity and external disturbances, the control architecture attempts to reduce this error.

Considering the presence of approximation error in fuzzy logic systems, the achievement of absolute asymptotic stability $\mathop {\lim }\nolimits_{t \to \infty } \left| {e\left( t \right)} \right| = \epsilon$ is practically challenging. The definition of the control objective is, therefore, modified to ensure uniformly ultimately bounded (UUB) stability. Mathematically, for any initial error $e\left( {t_{0} } \right)$, there exists a positive constant є and a finite time $T\left( {e\left( {t_{0} } \right),\epsilon } \right)$ such that:

17 $$\left| {e\left( t \right)} \right| \le \epsilon ,\quad \forall t \ge t_{0} + T$$

where $\epsilon$ error bound and *T* transient time.

This means that the tracking error will converge to and stay inside a limited, predetermined region of the origin, where $\epsilon$ is primarily governed by the fuzzy approximation error and the adaptation gain.

## Simulation results

A series of comprehensive simulations are carried out using the Pioneer 3-DX mobile robot model to verify the efficacy and resilience of the proposed self-tuning fuzzy PID (ST-FPID) control strategy. The velocity feedback linearisation controller suggested in Martins et al.^10^ and the traditional PID controller with fixed gains are the two control strategies used to compare the performance of the ST-FPID controller.

The DDMR’s dynamic parameters, which describe the robot’s physical characteristics and actuator restrictions, were established as follows: $\delta_{1} = 0.2604$, $\delta_{2} = 0.2509$, $\delta_{3} = - 0.000449$, $\delta_{4} = 0.9965$, $\delta_{5} = 0.00263$, $\delta_{6} = 1.0768$. Consistent with the experimental identification provided^10^. In all simulations the robot starts at position (0.2 0.0) m with orientation 0 degrees, and different trajectory tracking scenarios are used to verified the performance of the proposed control algorithm.

### Scenario 1: nominal conditions (ideal environment)

**Fig. 3:**
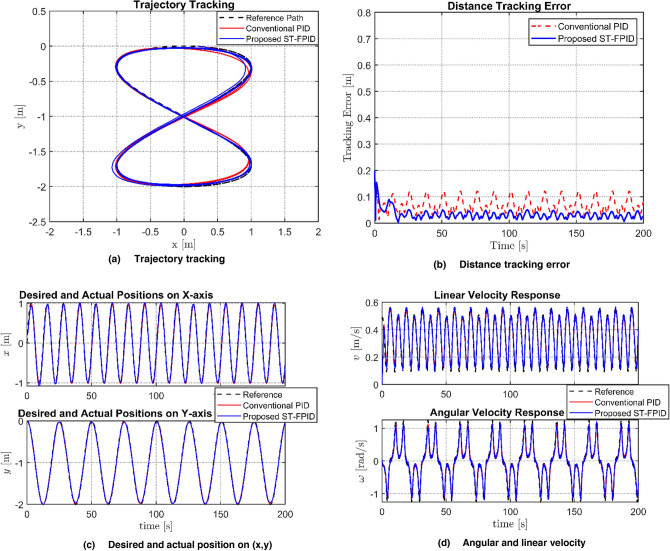
Performance comparison between PID and FPID controllers regarding: **a** Trajectory tracking, **b** Distance tracking error, **c** Desired and actual position **d** Agular and linear velocity.

The results of the first simulation scenario are shown in Fig. 3. This scenario represents an ideal case where the robot operates in a perfect environment, free from external disturbance. This scenario is used as a baseline to compare the efficacy of the proposed adaptive self-tuning fuzzy PID (ST-FPID) controller to the conventional PID controller.

The simulation results for first Scenario, as shown in Table 2 and Fig. 3, verify that the proposed ST-FPID controller is better than the conventional PID controller. The proposed controller reduced the ITAE and improved the accuracy by significantly reducing the value of the RMS error. These results, along with the rapid convergence of the system, as indicated by the plots of the errors, verify that the fuzzy system is able to minimize the tracking errors and maintain the system within the UUB region. As shown by the position plots, it is obvious that a near-perfect overlap is achieved by the proposed ST-FPID controller along both axes. From the linear and angular velocity profile plots, it is observed that the ST-FPID controller provides a much smoother control signal compared to the PID controller.

**Table 2:** Performance evaluation and comparison of tracking indices.

Controller	IAE (m s)	ITAE (m s^2^)	RMS
Conventional PID	12.03	1195	0.065
Proposed ST-FPID	7.2	663	0.04

### Scenario 2: robustness and disturbance rejection analysis

To critically assess the reliability and adaptability of the presented controller scheme, a second simulation test was conducted under a highly challenging environment. This test environment assesses the reliability of the controller in the presence of both internal model changes and external environment disturbances. This test simulated model uncertainties and parameter drift by purposefully mismatching the robot’s nominal physical parameters by 20%. The parameters were initialized as: ${\delta }_{1}$ = 0.3124, ${\delta }_{2}$ = 0.301, ${\delta }_{3}$ = − 0.000538, ${\delta }_{4}$ = 1.1958, ${\delta }_{5}$ = 0.00315, ${\delta }_{6}$ = 1.292. Moreover, the system was exposed to two different types of disturbances: Kinematic disturbance (${\zeta }_{kin}$): A random noise signal was added to the kinematic equations. The amplitude of the noise was set to 0.001.

Dynamic disturbance (${\zeta }_{dyn}$): A periodic external force was applied to the system and was given by the following sinusoidal equation: $d\left(t\right)=A \cdot {\sin}wt$ where the amplitude *A* = 1 and angular frequency *w* = 1 rad/s.

**Fig. 4:**
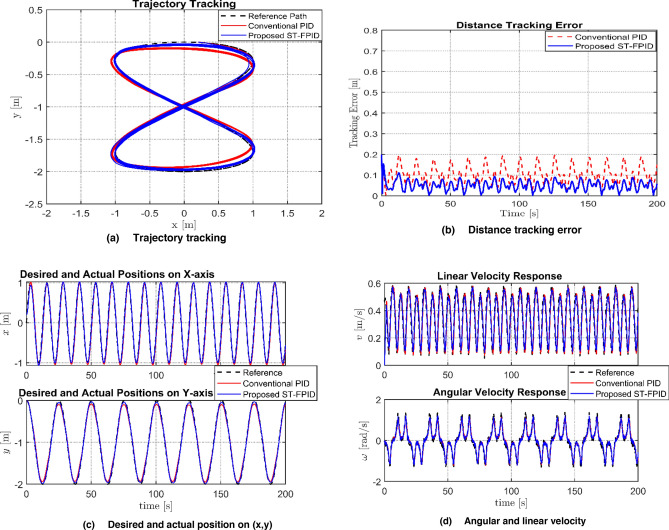
Performance comparison between PID and FPID controllers regarding: **a** Trajectory tracking, **b** Distance tracking error, **c** Desired and actual position **d** Agular and linear velocity.

The tracking performance of the proposed controller, namely ST-FPID, and conventional PID was tested under a 20% parameter mismatch condition and various sources of disturbances, including kinematic noise and sinusoidal dynamic forces. The graphical representation of this performance, as presented in Fig. 4, clearly indicates that the proposed controller is far superior in tracking performance with minimal phase lags. The PID controller is experiencing major oscillations due to the introduction of noise and sinusoidal dynamic forces, resulting in “chattering” of the velocity profile, whereas the proposed controller is able to effectively reduce these effects through its fuzzy adaptive mechanism, resulting in almost perfect synchronization of its velocity profile with the reference path of Lemniscate. As indicated in the Table 3, the performance of the proposed controller is far superior to that of PID in all statistical measures.

**Table 3:** Performance evaluation and comparison of tracking Indices.

Controller	IAE (m s)	ITAE (m s^2^)	RMS
Conventional PID	20.55	2058	0.1114
Proposed ST-FPID	10	976	0.0554

### Scenario 3: aggressive robustness test with severe parameter variation

To strictly test the limits of the proposed control scheme, a third simulation was performed with even more aggressive conditions. In this case, the physical robot model was deliberately mismatched by a substantial margin of 50% compared to the nominal robot parameters. Such severe robot physical model uncertainty was simulated by initializing the parameters as:${\delta }_{1}=0.3906$, ${\delta }_{2}=0.376$, ${\delta }_{3}=-0.0006735$, ${\delta }_{4}=1.4947$, ${\delta }_{5}=0.003945$, ${\delta }_{6}=1.6152$. . The system was subject to the same multi-source disturbances (kinematic noise and sinusoidal dynamic forces) as in Scenario 2. This test is a critical benchmark to evaluate whether the proposed ST-FPID can maintain trajectory integrity when the conventional PID faces excessive internal and external stress.

**Fig. 5:**
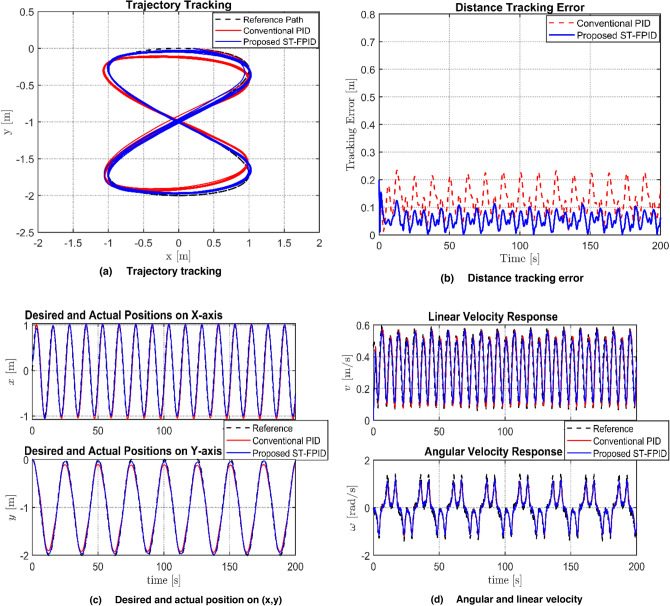
Performance comparison between PID and FPID controllers regarding: **a** Trajectory tracking, **b** Distance tracking error, **c** Desired and actual position **d** Agular and linear velocity.

The comparative results, as presented in Table 4 and Fig. 5, clearly indicate that the proposed ST-FPID has a definite edge over the traditional PID controller. Quantitatively, the suggested architecture has demonstrated a notable increase in the RMS tracking error reduction by 53.5%, from 0.1339 to 0.0622. Moreover, substantial improvements in the IAE and ITAE criteria have also been observed. Graphically, the proposed ST-FPID has shown high-precision tracking along the lemniscate path by effectively eliminating the deviation observed for the traditional PID controller during high-curvature points.

**Table 4:** Performance evaluation and comparison of tracking Indices.

Controller	IAE (m s)	ITAE (m s^2^)	RMS
Conventional PID	24.72	2471	0.1339
Proposed ST-FPID	11.25	1104	0.0622

### Scenario 4: ultimate stress and breakdown limit

Finally, to push the proposed control architecture to its absolute breaking point, a final stress test was performed. In this case, a 100% parameter mismatch was used, which means that a model was used where all of the robot’s dynamic parameters were completely inaccurate. The parameters were initialized with these perturbed values: ${\delta }_{1}=0.5208$, ${\delta }_{2}=0.5018$, ${\delta }_{3}=-0.000898$, ${\delta }_{4}=1.993$, ${\delta }_{5}=0.00526$, ${\delta }_{6}=2.1536$. In this case, just like in the previous ones, all the multi-source disturbances were active. This test is essential to determine the breaking point for each controller and to verify that even in the event of a critical system failure, the ST-FPID control method provides a safer response.

**Fig. 6:**
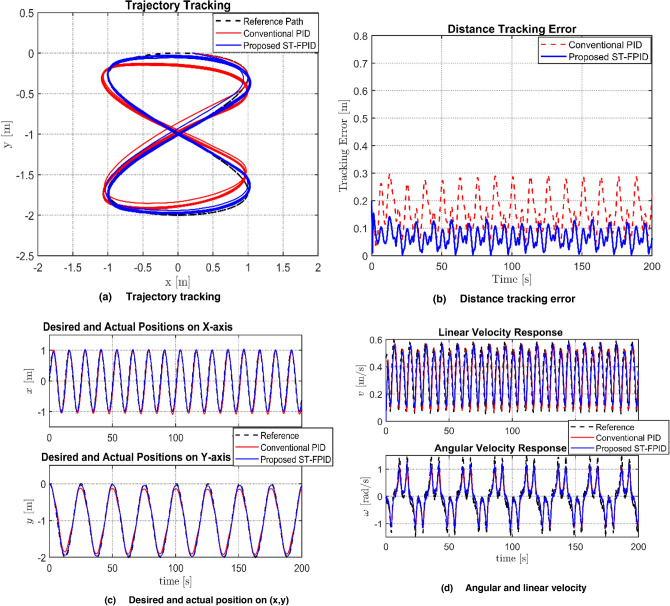
Performance comparison between PID and FPID controllers regarding: **a** Trajectory tracking, **b** Distance tracking error, **c** Desired and actual position **d** Agular and linear velocity.

The performance evaluation of the final scenario again emphasizes the strong capabilities of the proposed controller under the worst possible condition of operation. As presented in Table 5, it is clear that the analysis of this scenario also verifies that the performance indices are improved dramatically when compared to conventional PID control, where the value of the RMS error is decreased significantly by 56.4%, i.e., from 0.1717 to 0.0748, whereas the value of ITAE is decreased by almost 58%. The graphical analysis of this scenario, presented in Fig. 6, also verifies that the proposed controller is capable of eliminating initial errors as well as oscillations that appear during long-period operation, where it is clear that conventional PID control (red line) is significantly affected by stochastic noise, whereas the proposed controller (blue line) is able to maintain convergence to the reference trajectory of a lemniscate shape.

**Table 5:** Performance evaluation and comparison of tracking Indices.

Controller	IAE (m s)	ITAE (m s^2^)	RMS
Conventional PID	31.67	3152	0.1717
Proposed ST-FPID	13.5	1317	0.0748

## Conclusion

This study addressed the major issue of precise tracking control of mobile robots in unpredictable environments. A control structure based on an adaptive fuzzy gain scheduling PID controller was proposed to address the problems of conventional control methods. The proposed controller was rigorously tested under various complex situations, including a very challenging situation of a lemniscate ($\infty$) trajectory tracking under two type of disturbance and kinematic slip. The simulation results provided conclusive evidence of the controller’s performance in reducing the RMS tracking compared to conventional PID controllers. The major contributions of this study are presented in the following paragraphs:

- Verified robustness and stability: The proposed controller is verified for robustness and stability through uniformly ultimately bounded (UUB) stability analysis, which ensures that the error is guaranteed to converge through mathematical analysis, even under variations of up to 100% in system parameters and disturbances.
- Superior disturbance rejection: With the dynamic adaptation of the PID gains ($${k}_{p} ,{k}_{i} ,{k}_{d})$$, the system consistently performed better than the baseline controllers. This is evident from the tight error bound maintained by the system even under the influence of high-frequency actuator noise and external disturbances.
- Scalable practical framework: The contribution of the study is to bridge the gap between kinematic planning and dynamic execution while providing a computationally efficient solution for real-time high-precision navigation.

In summary, the contribution of the study is to advance the state of the art in the field of intelligent robotic control. The fuzzy gain scheduling approach would be validated experimentally on an actual robotic platform for future work to confirm its applicability to unstructured environments.

## Data Availability

All data generated or analyzed during this study are included in this published article.

## References

[1] Lee, D. H., Kim, Y. J. & Park, J. H. Development of an autonomous braking system using the predicted stopping distance. *Int. J. Autom. Technol.* **15**(2), 341–346 (2014).

[2] Yang, J. & Coughlin, J. F. In-vehicle technology for self-driving cars: Advantages and challenges for aging drivers. *Int. J. Autom. Technol.* **15**(2), 333–340 (2014).

[3] Amoozgar, M. H. A fuzzy logic-based formation controller for wheeled mobile robots. *Ind. Robot.* **38**(3), 269–281 (2011).

[4] Peng, J., Yu, J. & Wang, J. Robust adaptive tracking control for nonholonomic mobile manipulator with uncertainties. *ISA Trans.* **53**(4), 1035–1043 (2014). 24917071 10.1016/j.isatra.2014.05.012

[5] Normey-Rico, J. E., Gómez-Ortega, J. & Camacho, E. F. Mobile robot path tracking using a robust PID controller. *Control Eng. Pract.* **9**(11), 1209–1214 (2001).

[6] Resende, C. Z., Carelli, R. & Sarcinelli-Filho, M. A nonlinear trajectory tracking controller for mobile robots with velocity limitation via fuzzy gains. *Control Eng. Pract.* **21**(10), 1302–1309 (2013).

[7] Khan, H., Iqbal, J. & Islam, R. U. Longitudinal and lateral slip control of autonomous wheeled mobile robot for trajectory tracking. *Front. Inf. Technol. Electron. Eng.* **16**(2), 166–172 (2015).

[8] Ribeiro, T. T. & Conceição, A. G. S. Nonlinear model predictive visual path following control to autonomous mobile robots. *J. Intell. Robot. Syst.* **95**(2), 731–743 (2019).

[9] Kayacan, E. & Chowdhary, G. Tracking error learning control for precise mobile robot path tracking in outdoor environment. *J. Intell. Robot. Syst.* **95**, 975–986 (2019).

[10] Martins, F. N., Sarcinelli-Filho, M. & Carelli, R. A velocity-based dynamic model and its properties for differential drive mobile robots. *J. Intell. Robot. Syst.* **85**(2), 277–292 (2017).

[11] Wu, X., Jin, P., Zou, T., Li, H. & Wang, Y. Backstepping trajectory tracking based on fuzzy sliding mode control for differential mobile robots. *J. Intell. Robot. Syst.* **96**, 109–121 (2019).

[12] De La Cruz, C. C. & Carelli, R. Dynamic modeling and centralized formation control of mobile robots. In *Proceedings of the 32nd Annual Conference of the IEEE Industrial Electronics Society (IECON), Paris, France*, 3880–3885 (2006).

[13] Taheri-Kalani, J. & Zarei, N. An adaptive technique for trajectory tracking control of a wheeled mobile robot without velocity measurements. *Autom. Control Comput. Sci.* **50**(6), 441–452 (2016).

[14] Cho, S., Shrestha, B., Jang, W. & Kim, J. Trajectory tracking optimization of mobile robot using artificial immune system. *Multimed. Tools Appl.* **78**, 3203–3220 (2019).

[15] Wang, J., Zhang, H., Li, Y. & Chen, X. (2011). An adaptive trajectory tracking control of wheeled mobile robots. In *Proceedings of the 6th IEEE Conference on Industrial Electronics and Applications (ICIEA 2011)*, pp. 1–6. IEEE.

[16] Zhao, P., Li, X., Sun, Y. & Chen, L. Design of a control system for an autonomous vehicle based on adaptive-PID. *Int. J. Adv. Robot. Syst.* **9**(2), 44 (2012).

[17] Antonini, P., Ippoliti, G. & Longhi, S. Learning control of mobile robots using a multiprocessor system. *Control Eng. Pract.* **14**, 1279–1295 (2006).

[18] De La Cruz, C. & Carelli, R. Dynamic model based formation control and obstacle avoidance of multi-robot systems. *Robotica* **26**(3), 345–356 (2008).

[19] Chen, C., Li, T., Yeh, Y. & Chang, C. Design and implementation of an adaptive sliding-mode dynamic controller for wheeled mobile robots. *Mechatronics* **19**(2), 156–166 (2009).

[20] De La Cruz, C., Celeste, W. & Bastos-Filho, T. A robust navigation system for robotic wheelchairs. *Control Eng. Pract.* **19**(6), 575–590 (2011).

[21] Rossomando, F. G., Soria, C. & Carelli, R. Adaptive neural sliding mode compensator for a class of nonlinear systems with unmodeled uncertainties. *Eng. Appl. Artif. Intell.* **26**(10), 2251–2259 (2013).

[22] Utstumo, T., Berge, T. W. & Gravdahl, J. T. Non-linear model predictive control for constrained robot navigation in row crops. In *Proceedings of the IEEE International Conference on Industrial Technology (ICIT 2015)*, pp. 1–8 (IEEE, 2015).

[23] Munoz, J., Copaci, D. S., Monje, C. A., Blanco, D. & Balaguer, C. Iso-m based adaptive fractional order control with application to a soft robotic neck. *IEEE Access* **8**, 198964–198976 (2020).

[24] Visioli, A. & Legnani, G. On the trajectory tracking control of industrial SCARA robot manipulators. *IEEE Trans. Ind. Electron.* **49**(1), 224–232 (2002).

[25] Hassan, N. & Saleem, A. Neural network-based adaptive controller for trajectory tracking of wheeled mobile robots. *IEEE Access* **10**, 13582–13597 (2022).

